# Synthesis of (Hyper)Branched Monohydroxyl Alkoxysilane Oligomers toward Silanized Urethane Prepolymers

**DOI:** 10.3390/molecules27092790

**Published:** 2022-04-27

**Authors:** Sebastian Kowalczyk, Maciej Dębowski, Anna Iuliano, Sebastian Brzeski, Andrzej Plichta

**Affiliations:** Chair of Chemistry and Technology of Polymers, Faculty of Chemistry, Warsaw University of Technology, Noakowskiego 3, 00-664 Warsaw, Poland; maciej.debowski@pw.edu.pl (M.D.); anna.iuliano@pw.edu.pl (A.I.); sebastian.brzeski.dokt@pw.edu.pl (S.B.); andrzej.plichta@pw.edu.pl (A.P.)

**Keywords:** hyperbranched oligomers, silane, polyurethane, urethane prepolymers, silanized polyurethane, 3-aminopropyl trialkoxysilane, ethylene carbonate, hydroxyl-terminated trialkoxysilane, condensation

## Abstract

The aim of this work was the synthesis of (hyper)branched oligomers based on trialkoxysilane in various conditions and further application of them in order to modify the urethane prepolymers. Hydroxyl-terminated trialkoxysilane was used as a monomer for homo-condensation. It was obtained by reaction of 3-aminopropyl trialkoxysilane (APTES) with ethylene carbonate (EC). The reaction was based on the attack of amine at the carbonyl carbon atom followed by ring opening of the carbonate to give a urethane (carbamate) product. The next step was the condensation via substitution of ethoxy groups on silicon atom with the terminal hydroxyalkyl groups present in the primary product with the evolution of ethanol. Accordingly, the impact of temperature and type of catalyst on process efficiency was investigated. A quantitative analysis of reaction progress and products of the conversion of EC together with ethanol evolution was conducted by means of gas chromatography, which allowed us to determine the formation of monomeric product and, indirectly, of oligomeric products. It was found that at room temperature after 24 h, the majority of the monomeric product was isolated, whereas at elevated temperature in the presence of Ti-based catalyst, further condensation of the monomer into branched oligomers was preferred, and, moreover, the application of vacuum intensified that process. The obtained products were structurally characterized by ^1^H and ^29^Si NMR, MALDI-ToF and Gel Permeation Chromatography. Finally, two different alkoxysilane products, monomeric and oligomeric, were applied for modification of urethane prepolymer, forming silanized one (SPUR). The influence of the silanizing agent on the mechanical and thermal properties of the moisture-cured products was shown before and after additional conditioning in water.

## 1. Introduction

Silicon polymers, due to human friendliness and universal properties, constitute a wide group of various materials and have been used in many areas of everyday life. For instance, due to their excellent chemical, physical and electrical properties, they are widely used in the food and cosmetics industry [1,2,3], medicine [4,5,6], electronics [7,8,9,10], building construction and as adhesion promoters [11,12,13]. The most common polymer of this kind is polydimethylsiloxane (PDMS), having a backbone consisting of siloxane groups (Si-O-Si) with two methyl groups on each silicon atom. Polysiloxanes are obtained in a three-step synthesis. The substrates are elemental Si (obtained by high temperature reduction in silicon dioxide (quartz or silica)) and methyl chloride. The products of direct Rochow’s process are chlorosilanes [14,15,16,17]. In the next step, they undergo hydrolysis to form linear or cyclic dimethylsiloxane oligomers, which in the final stage are subjected to, respectively, polycondensation or chain polymerization, catalyzed by acids or bases. Due to the lack of diversity in structure and properties, there is a great need to modify polysiloxanes by introducing various functional groups into their structure. An example of such a modification is the introduction of functional groups at the synthesis stage: azide groups (capable of 1,3-dipolar cycloaddition reactions), vinyl groups (giving many pathways of modification, including hydrosilylation or thiol-ene reaction) or amine groups (capable of Michael addition). Such activities are aimed at improving individual properties of materials and creating a range of materials for new applications [11,18,19,20,21].

**Figure 1:**
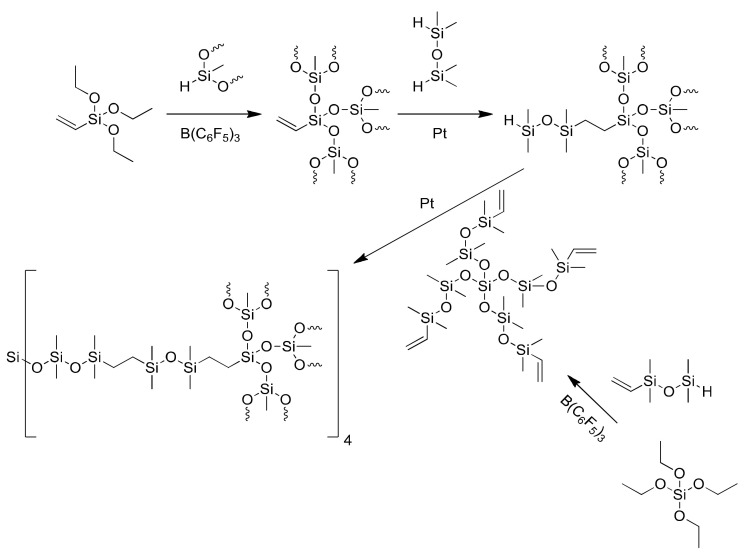
An exemplary synthesis of a hyperbranched siloxane using the Piers–Rubinsztajn reaction and hydrosilylation process.

Over the past decades, dendritic polymers, particularly dendrimers and hyperbranched polymers, have attracted extraordinary interest. This class of macromolecules exhibits unique, architecture-correlated properties. Among others, the properties include: unusually low dispersities of molar masses, Newtonian fluid characteristics even at very high molar mass, encapsulation abilities in host–guest interactions, as well as much smaller hydrodynamic volumes, much more compact molecules, much higher solubilities and much lower viscosities than their corresponding linear analogs of the same molar mass [22,23]. Researchers have also focused on the synthesis of silicon-based hyperbranched polymers prepared with a relatively narrow set of reactions, such as hydrosilylation, Grignard reactions and controlled condensation of silanols. Complete conversion of all functional groups is necessary for creating well-defined dendrimer molecules; however, it might be a challenge due to a large number of functional groups at one macromolecule, which must undergo reaction at the same time (generation) [24]. The Piers–Rubinsztajn reaction for the preparation of siloxane, carbosilane and carbosiloxane hyperbranched structures is extensively utilized. Basically, monohydrotrisiloxanes (e.g., Heptamethyltrisiloxane) react with trialkoxyvinylsilanes; then, branched siloxanes and low molar mass alkanes are generated in the presence of BCF (B(C_6_F_5_)_3_) as a catalyst [25,26,27,28,29,30,31,32,33], whereas in order to obtain carbosiloxanes, the other type of reaction has to be applied (Figure 1). It can include hydrosilylation process, for instance, which is based on the addition reaction between unsaturated bonds (e.g., CH=CH_2_) and silicon−hydrogen (Si−H) bonds in the presence of a platinum complex catalyst [34,35,36].

Another group are dendrimers, consisting of polyhedral oligomeric silsesquioxanes (POSS) as a core, obtained utilizing various techniques, depending on the groups introduced into POSS core. Recently, POSS molecules have attracted a lot of interest in chemistry. Their use as a core of a dendrimer has some advantages. The POSS molecule is cube shaped, with functional groups located at each vertex, giving eight sites where a dendrimer can develop. These sites can be synthetically modified to contain groups capable of a variety of reactions. Most dendrimers are based on tetravinylsilane, tetraallylsilane, pentaerythritol or propyl amino compounds [22,37,38,39,40,41,42].

One of the silicon-based chemical compounds, which is widely utilized in chemical reactions leading to siloxane polymers or silane modified (macro) molecules or particles, is 3-aminopropyl trialkoxysilane (APTES). A total of 15,745 scientific articles and patents concerning the use of APTES in the synthesis of compounds and preparation of materials have been published since 1968, while from 2020, only journal publications in English amount to 1287 items [43]. The main areas of application of APTES include synthesis and modification of (nano) particles and surfaces with amine functionality on the outsphere, allowing further reactions, such as conjugation with polymers, drugs, radiotherapeutics, imaging agents [44], catalysts, anisotropic carbon nanomaterials, metal ion scavengers, antimicrobial agents and many others [45,46,47]. APTES is also applied for various polymers’ modification, e.g., silanization of urethane prepolymers or epoxy resins [48].

**Figure 2:**
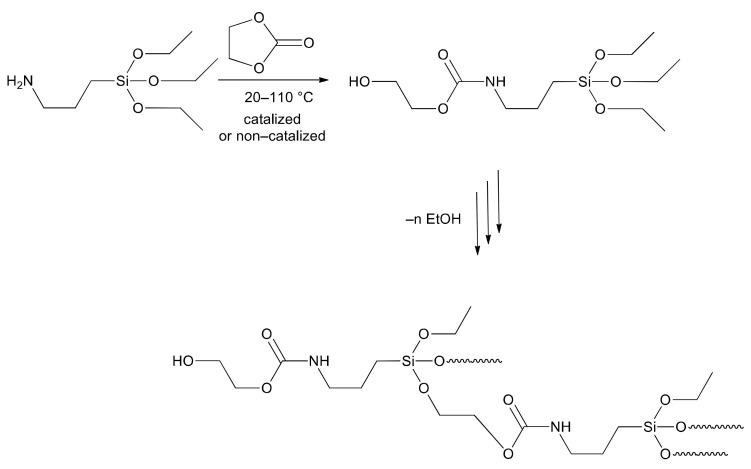
Scheme of the synthesis of APTES-OH monomer and its (hyper) branched oligomers terminated with a hydroxyl group.

The aim of this work was the synthesis of (hyper) branched oligomers based on trialkoxysilane in various conditions and further application of them in order to modify the urethane prepolymers. Hydroxyl-terminated trialkoxysilane was used as a monomer for homo-condensation. It was obtained by reaction of APTES with ethylene carbonate (EC). The reaction is known from the literature [22,49,50], and it is based on the attack of amine at the carbonyl carbon atom, followed by ring opening of the carbonate to give a urethane (carbamate) product (Figure 2). The next step was the condensation via substitution of ethoxy groups on silicon atom, with the terminal hydroxyalkyl groups present in the primary product with the evolution of ethanol (Figure 2). Accordingly, the impact of temperature and type of catalyst on process efficiency was investigated. Finally, two different alkoxysilane oligomers were applied for the modification of urethane prepolymer (PUR) forming silanized one (SPUR). The influence of the silanizing agent on the properties (mechanical, thermal) of the obtained products is shown.

## 2. Results and Discussion

### 2.1. Influence of Temperature and Catalysts on the Degree of Polymerization of Hydroxyl-Terminated Alkoxysilane Oligomers

In order to obtain hydroxyl-terminated alkoxysilane monomer and oligomers, the reactions of a primary amine group of APTES with a five-membered EC were exploited in this work. The reaction proceeds via nucleophilic attack of amine on the carbonyl group in cyclic carbonate structure followed by ring opening of the carbonate to give a product comprising carbamate moiety (Figure 2). This type of reaction is well known in the literature and is also widely used for the synthesis of non-isocyanate PUR [51,52,53]. However, the nascent product of the reaction of APTES with EC contains a primary hydroxyl group and three ethoxysilane bonds in each molecule; therefore, it may undergo a consequent reaction based on the exchange of alkoxy substituents on silicon atoms, i.e., ethoxy with APTES-OH. The polycondensation process of the APTES-OH monomer takes place under favorable conditions, which may lead to the mixture of linear and (hyper) branched oligomers as presented in Figure 2.

For the purpose of the screening of catalysts and conditions that influence the structure of products obtained, the equimolar amounts of the reactants were used, and the reactions were carried out in bulk, without a solvent (1.5 wt% of toluene or xylene was used as an internal standard for GC analysis), without a catalyst at temperatures of 20, 30, 50, 70, 90 and 110 °C and additionally, with 0.5 wt% of catalyst based on: Sn(II), Sn(IV), Zr(II), Zn(II) and Ti(IV) compounds, as well as following organocatalysts, DABCO, DBU, TEA and Ph_3_P, at temperatures of 30 and 50 °C. The list of performed reactions is presented in Table 1.

**Figure 3:**
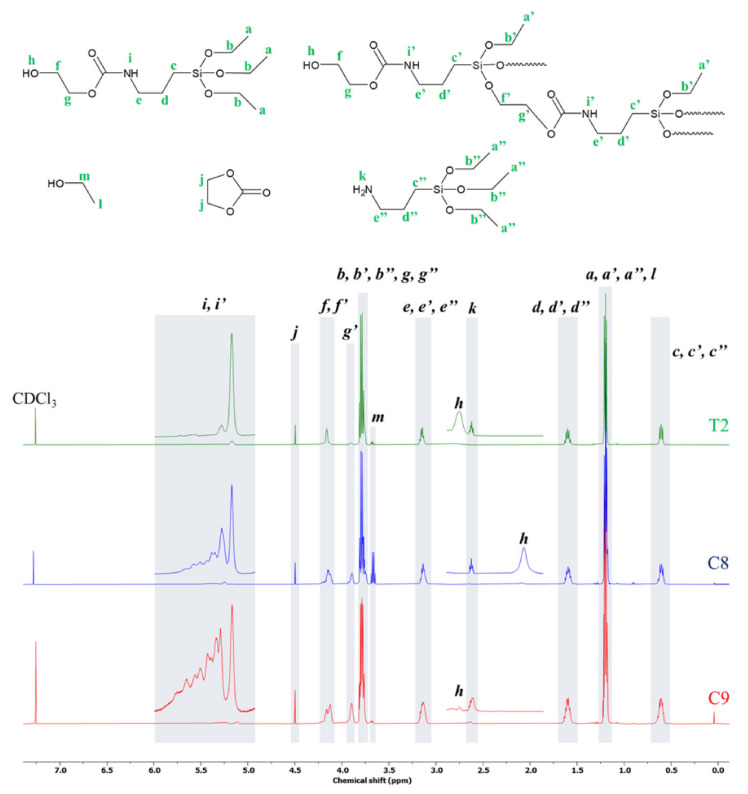
^1^H NMR spectrum of products in CDCl_3_.

The ^1^H NMR spectra of the products T2 (25 °C, no catalyst), C8 (50 °C, Ti(IV) catalyst) and C9 (50 °C, Ti(IV) catalyst, vacuum) are shown in Figure 3. The spectrum of T2 (a) reveals signals for methyl (a, 1.2 ppm) and methylene (b, 3.8 ppm) protons in the oxyethylene groups bond to silicon as well as a signal of methylene protons at carbon bond directly to silicon (c, 0.6 ppm) and following methylene groups in propylene chain (d, 1.6 ppm and e, 3.15 ppm). The confirmation of monomeric product structure may be read from the signals of chemical shift at δ 4.15 and 3.8 ppm, corresponding to the protons in methylene groups next to oxygen atoms of the formed urethane group (f and f’—in monomer and oligomer structure, respectively) and also hydroxyl group (g, overlapping with signal b), respectively. Moreover, the signals of protons bond to oxygen atoms in hydroxyl (h, 2.78 ppm) and to nitrogen in carbamate (i, 5.18 ppm) groups, correspondingly, are seen clearly. Additionally, there are minute signals at δ 4.5 (j) and 2.62 ppm (k) revealing some small amounts of residual EC and APTES. When comparing the ^1^H NMR spectrum of T2 with the ones of C8 and C9, one can see that increasing temperature, the introduction of catalysts and arbitrary application of vacuum in order to remove polycondensation by-product (ethanol, l and m), resulted in some changes in products structure. For instance, there was a separation of signals from methylene (b, 3.8 ppm) protons in oxyethylene groups and the signals from methylene groups next to oxygen atoms of the hydroxyl group (g’, 3.9 ppm). In addition, the signals of protons bond to oxygen and nitrogen atoms in carbamate (i, 5.18 ppm) groups became more extensive.

**Figure 4:**
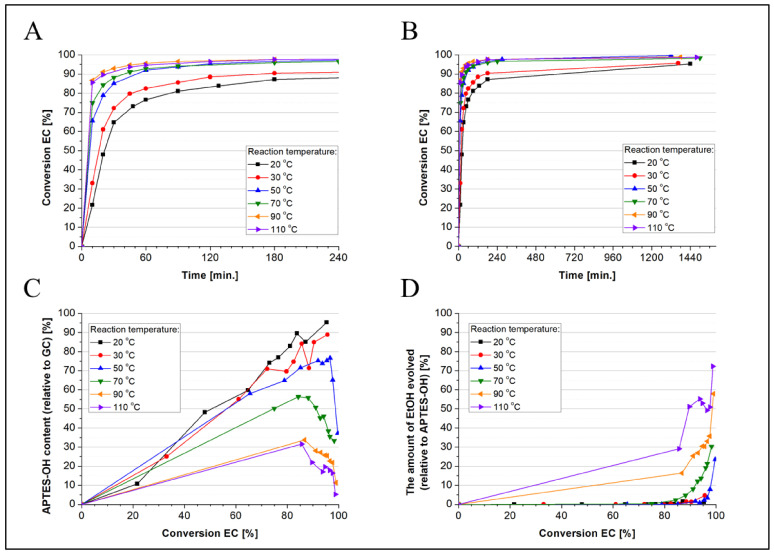
The conversion of EC during non-catalyzed synthesis of APTES-OH depending on the reaction temperature (**A**,**B**), the content of APTES-OH (**C**) and ethanol (**D**) in relation to the conversion of EC depending on the reaction temperature.

**Figure 5:**
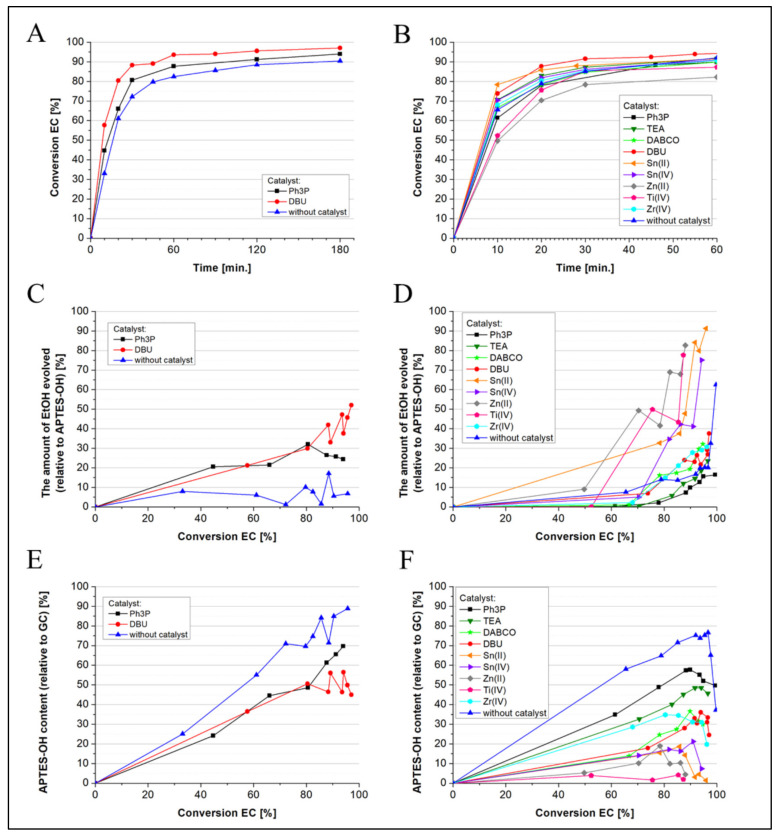
The conversion of EC (at 30 °C for (**A**) and 50 °C for (**B**)) during the reaction depending on the catalyst used, the APTES-OH content (at 30 °C for (**C**) and 50 °C for (**D**)) depending on the catalyst and the amount of ethanol (at 30 °C for (**E**) and 50 °C for (**F**)) released in relation to the EC conversion.

For a quantitative analysis of reaction progress and products, the conversion of EC together with ethanol evolution was measured by means of GC, which allowed us to determine the formation of, respectively, the main monomeric product (APTES-OH) and oligomeric side products. It gives some correlation between the reaction rate and selectivity. The results plotted over time are shown in Figure 4 and Figure 5. It was found that with no catalyst under rising temperature, the rate of EC conversion increased significantly; however, it also produced a large amount of by-products resulting from the condensation of the hydroxyl monomer (Figure 4). When analyzing the EC conversion, it was noticed that at 20 °C after 20 min, the EC conversion was 47.9%, and the content of ethanol was 0%, while after 24 h, it was 95.3% and just 1.13%, respectively. On the other hand, at 110 °C, these values were 89.6% and 51.1% after 20 min, and also 98.7% and 72.3% after 24 h, accordingly. Comparing all the results, it can be concluded that the APTES-OH synthesis process runs rather slowly but selectively at low temperatures up to 30 °C. On the other hand, if vigorous oligomerization of APTES-OH is necessary, a temperature above 50 °C should be applied, so the conversion of the reactants is much faster, and the condensation reaction becomes significant. The siloxane exchange of the ethoxy groups with the hydroxyl groups of the monomer APTES-OH or its hydroxyl oligomeric derivatives, which leads to the production of ethanol and condensation products, occurs satisfactorily at higher temperatures.

When organic catalysts were introduced to these processes carried out at 30 °C, it slightly increased the EC conversion rate (especially for DBU), whereas at 50 °C, in the case of metal-based and organic catalysts, the differences were negligible and, moreover, for Ti(IV), Zn(II) and Ph_3_P systems, the conversion rate was even lower. However, in all these processes, the presence of a catalyst accelerated moderately or significantly the reaction of APTES-OH condensation. It is worth noting that at 50 °C, when using catalysts based on Sn(II), Zn(II), or Ti(IV), the APTES-OH content did not exceed 20% (for Ti(IV), it did not exceed 5%) in any studied EC conversion, although the latter one finally reached above 85%. At the same time, the amount of released EtOH increased rapidly in these systems; therefore, the listed catalysts (principally Ti(IV)) appear to be the most effective for the synthesis of APTES-OH oligomers. DBU, TEA and Ph_3_P catalysts are not so effective in the formation of condensation products. In summary, the best conditions to obtain a monomeric product (APTES-OH) are to carry out the reaction at a temperature of 20–25 °C, without the use of a catalyst. On the other hand, in order to obtain the APTES-OH oligomers with the greatest efficiency, the reaction should be carried out at an elevated temperature (50–90 °C) with the use of Ti(IV) as a catalyst. For this reason, it was also decided to carry out a synthesis using the Ti(IV) catalyst and to collect the low-molecular-weight EtOH by-product through vacuum distillation for 3.5 h. The analysis results obtained for this sample are presented in the further course of the work. The conversion of EC and the APTES-OH content during the reaction depending on the catalyst used as well as the amount of ethanol released in relation to the EC conversion are shown in Figure 5. One can say that the alcoholysis of the Si–OEt bond in APTES-OH and its oligomers by hydroxyl group contained in them is the transesterification process. These kinds of processes are sufficiently catalyzed by Ti(IV) alkoxides, e.g., in the case of PET synthesis. Here, we would expect that the catalytic effect of Ti(IV) alkoxide is caused by its coordinate unsaturation, the electrophilicity of metal and the electron-withdrawing effect of the O–Ti bond in the Si=(O)_2_=Ti complex comprising APTES-O-Ti and APTES-OH (Si-OEt bond) molecules. Titanium tetraalcoholates exhibited high catalytic activity in Si-OEt hydrolysis shown by Katayama et al. [54].

**Figure 6:**
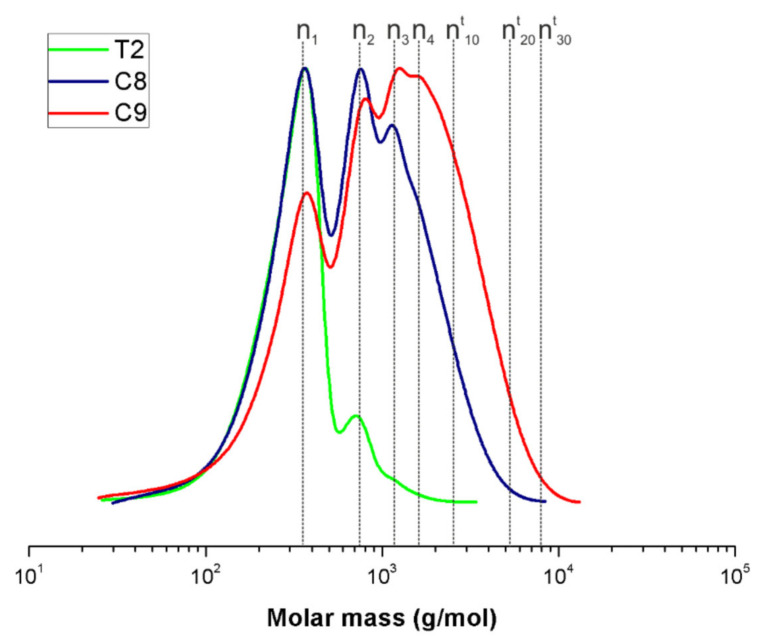
GPC molar mass distribution for T2 (25 °C, without catalyst), C8 (50 °C, Ti(IV)) and C9 (50 °C, Ti(IV), vacuum) products (n^t^_x_—theoretical value of the molar mass for a particular number of x units).

**Figure 7:**
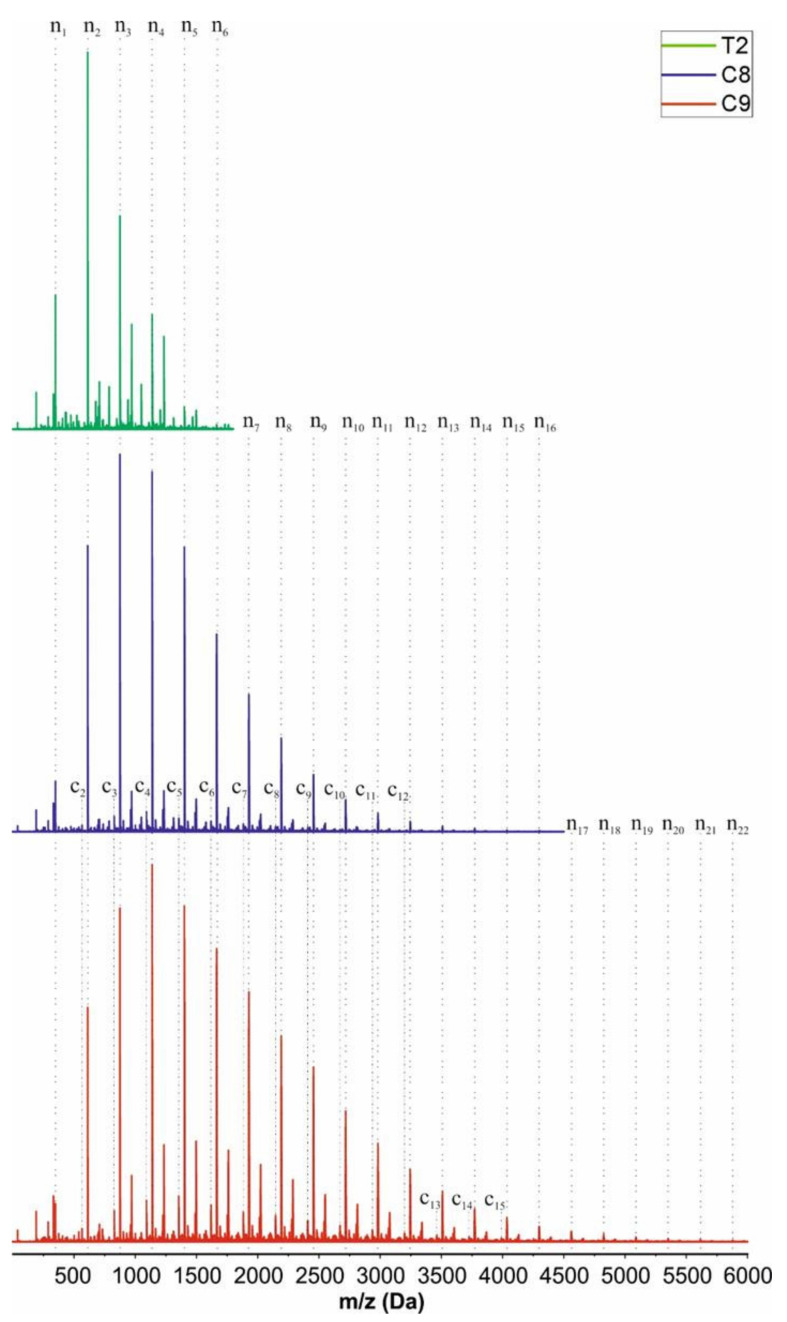
MALDI-ToF analysis for T2 (RT, without catalyst), C8 (50 °C, Ti(IV)) and C9 (50 °C, Ti(IV), vacuum) products.

The formation of monomer and oligomeric structures is confirmed by GPC (Figure 6) and MALDI-ToF analyses (Figure 7). Molar mass distribution given by GPC reveals that APTES-OH monomer was obtained with satisfactory yield (88%) and selectivity in the case of sample T2 (25 °C, no catalyst). There are just small amounts of condensation products identified principally as dimers and trimers. On the other hand, when the Ti(VI)-based catalyst was used, and the temperature was elevated in the process up to 50 °C (C8), the complex, multimodal molar mass distribution was exhibited, and M_n_ of the product increased significantly, and thus, its ability to form oligomeric (branched) structures. Moreover, the condensation equilibrium was shifted toward higher oligomers (M_n_ 2500 g/mol), while vacuum distillation of the by-product was applied to the system (C9). 

The results of the MALDI-ToF analysis confirmed the conclusions drawn previously for the C8 (50 °C, Ti(IV)) and C9 (+vacuum) products, and it showed the m/z signals belonging to monomer (n1; minor) and oligomers comprising up to 16 and 22 monomeric units, respectively. The main population (Supplementary Materials Figure S1, structure S1.1) turned out to be the linear and/or branched oligomer formed during condensation, with the repeating unit of 263.12 g/mol flying with K+ ions, m/z of end group 46.51 g/mol (the distribution indicated with n_x_ in Figure 7). The other existing but much less intensive population is characterized by the same *m*/*z* value of the repeating units but with a different mass of end groups of 142.29 g/mol. The intensity of this population increases when the process conditions become more severe. However, the authors of this paper were unable to assign any structure to it. The possible structures formed in the following reactions considered were: attachment of two EC molecules to one APTES molecule, formation of a partial network of Si-O-Si bonds, intramolecular cyclization with the release of EC or ethylene glycol, etc., presented in Figure S1 (structure S1.3–S1.9). The repeat units and the masses of the end groups of the proposed structures are listed in Table S1. Additionally, for all products examined (T2, C8 and C9), one can find a population of very low intensity resulting from macrocycles generated in the process of intramolecular condensation, with the repeating unit of 263.12 g/mol fluing with K^+^ ions, *m*/*z* of end group 0.37 g/mol (the distribution is marked with cx in Figure 7). The structure is shown in Figure S1 (structure S1.2). Surprisingly, for sample T2, the signals for the monomer, dimer, trimer and tetramer are present on MALDI-ToF analysis, but the one for the dimer is the most intensive, whereas APTES-OH monomer was the major product indicated by GPC analysis of the concerned sample. Although the MALDI ToF analysis is not quantitative, it should be expected that APTES-OH monomer molecules with the lowest molar mass, and thus most willingly flying under the measurement conditions, which we would expect the most in the T2 sample, should be visible on the spectrum in the signal with the highest intensity. It was suspected that before or during the MALDI ToF experiment, the sample partially underwent some undesirable processes (e.g., monomer distillation or condensation driven by a high vacuum in the instrument chamber). In order to verify this hypothesis, the esterification reaction of the T2 product with oleic acid in the presence of dicyclohexyl carbodiimide was carried out, resulting in the derivative T2_OAE_, which was not able to self-condense and was much less volatile.

**Figure 8:**
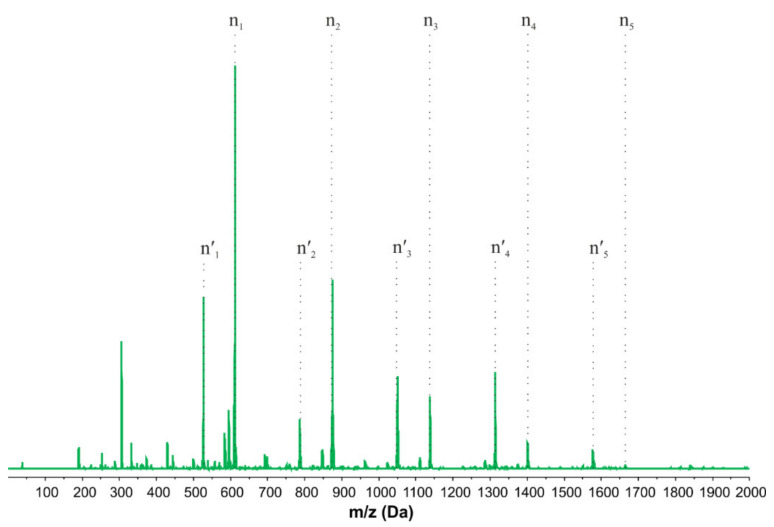
MALDI-ToF analysis of the reaction product of T2 with oleic acid.

When analyzing the obtained MALDI-ToF result for the T2_OAE_ sample (Figure 8), the absence of T2 and its oligomers can be confirmed (no signals from unesterified monomer, dimer, trimer, etc.), while signals of the corresponding oleic acid esters were present (n_x_ distribution, *m*/*z* of repeating unit of 263.12 g/mol, *m*/*z* of end group 310.27 g/mol). In addition, there is a minor distribution from the population of a similar structure, in which the release of one EC molecule contained in the end group occurred during the reaction of the APTES-OH monomer and oligomers with oleic acid (n^′^_x_ distribution, *m*/*z* of repeating unit of 263.12 g/mol, *m*/*z* of end group 222.30 g/mol, which is 88 g/mol less than for n_x_ population), forming an amide bond instead of an ester one. Additionally, the signal derived from the ester with APTES-OH monomer revealed the highest intensity in the mass spectrum, which correlates with ^1^H NMR and GPC results, so it can be assumed that the conditions of MALDI-ToF analysis favor the conversion of APTES-OH monomer into oligomers.

**Figure 9:**
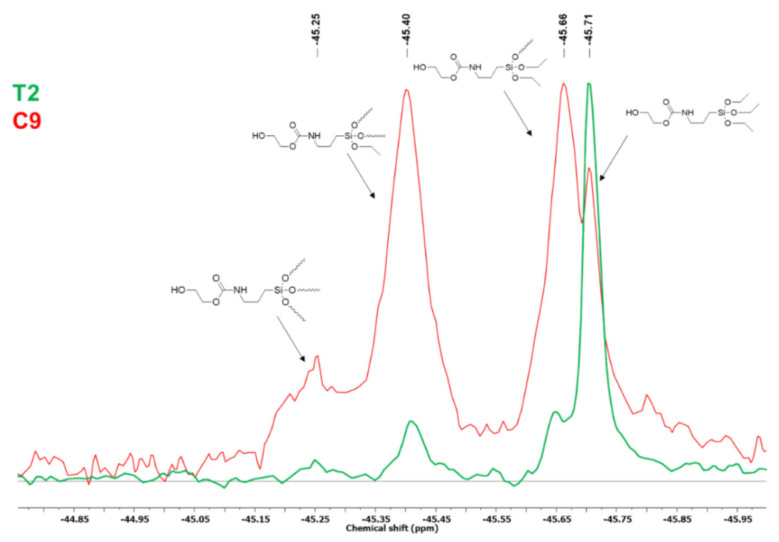
^29^Si NMR spectrum of products T2 (RT, without catalyst) and C9 (50 °C, Ti(IV), vacuum).

The architecture of the obtained oligomers, including the type of branching, was investigated by means of ^29^Si NMR (Figure 9). Four resonance peaks in the range of −45.75 ÷ −45.25 ppm are visible on the spectra. The resonance peak of the chemical shift of 45.71 ppm, which was predominant in the case of the T2 sample, which was rich in APTES-OH monomer, is related to a silicon atom with three ethoxy groups attached. It might be attributed to the monomer molecules, as well as to the end monomeric unit of the oligomer. The other significant second signals present in the T9 (vacuum condensation product) spectrum at a chemical shift of −45.66 and −45.40 ppm correspond to the silicon atoms bonded to two and one ethoxy groups, respectively, whereas the minor peak at −45.25 ppm most likely represents the Si atom without such substituent. The presence of numerous Si atoms comprising some or all ethoxy moieties replaced with other alkoxy groups coupled via carbosilane bonds to another Si(OR)_3_ species proves that the obtained silylsiloxane oligomers are (hyper) branched.

### 2.2. Synthesis of SPUR

**Figure 10:**
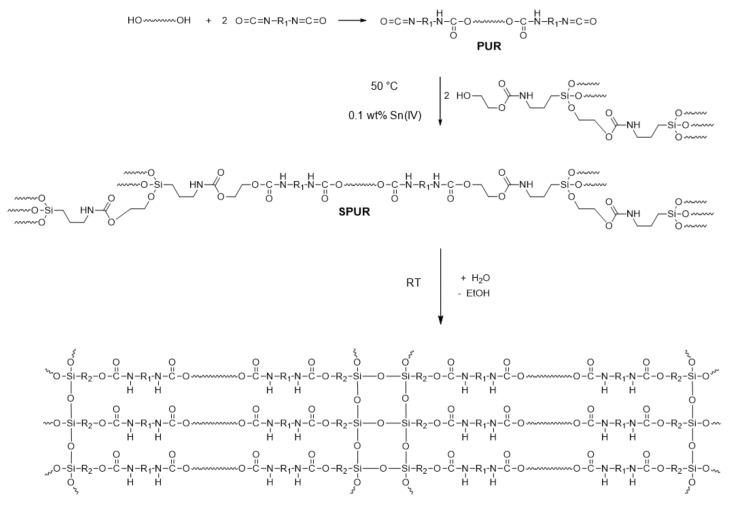
Synthesis of SPUR and cross-linking scheme.

**Figure 11:**
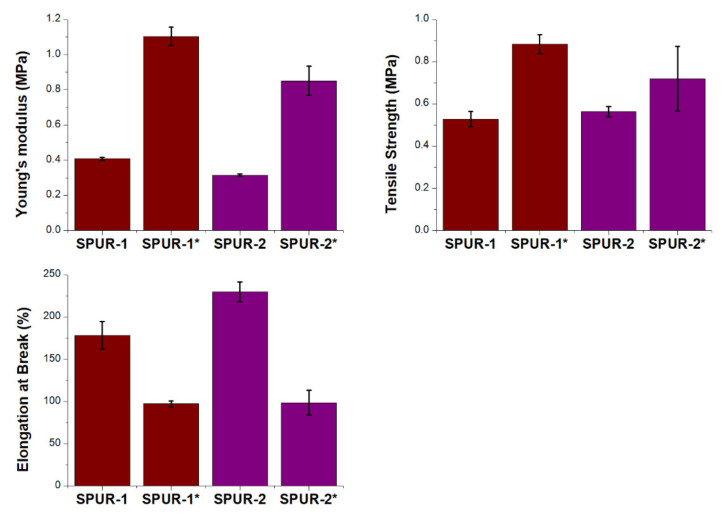
Mechanical strength results for SPUR samples. (* samples after aging, 4 weeks in water).

Two different products of the reaction of APTES with EC, i.e., T2 and C9, which mainly comprised the monomer or branched oligomer, respectively, were used as monohydroxyl silanizing agents for PUR based on oligoetherols. PUR was synthesized by reacting PETOL with TDI at the molar ratio of 1:2, in order to assure isocyanate end groups and possibly the low molar mass of the product (Figure 10). Then, isocyanate moieties were utilized for the reactions with T2 or C9 (at an equal molar ratio of -OH/-NCO), which led to the products, SPUR-1 and SPUR-2, accordingly. The course of the reactions was monitored by FTIR (Figure S2), observing the complete disappearance of the bands originating from the isocyanate group (2270 cm^−1^) and the rising of the band attributed to the vibrations of N–H in urethane groups (3330 cm^−1^). Each formulation contained 20 wt% of DINP applied as a low-volatile solvent. Finally, the two obtained SPUR compositions able to cross-link in the presence of water vapors via Si-OR hydrolysis and the formation of siloxane bonds (Figure 11) were poured onto a PTFE surface to form a thin (1–2 mm) cured polymer film.

The tensile properties of SPUR were determined for the resulting materials (Table S2). The tensile strength value reached a bit more than 500 kPa in the case of both samples studied; however, SPUR-2 exhibited a slightly higher value. The elongation at the break is almost 30% higher for the SPUR-2 sample (almost 230%) when compared to SPUR-1. The SPUR-2 sample showed higher flexibility as well. This gives the conclusion that higher oligomerization degree and branching degree of the presented silanizing agent caused improvement in flexibility and extensibility of SPUR at no significant change in tensile strength. It is somehow surprising because one could expect that SPUR-2 would reveal higher strength and rigidness but a lower ability to elongate because of the more dense network obtained in cross-linking the prepolymers of higher content of functional groups per molecule. The results might suggest that not all of the Si-OEt groups underwent hydrolysis and further condensation. Additionally, the hydrolytic stability of the samples was tested by immersing them in water for 4 weeks at room temperature. The obtained results are presented in Table S2 and Figure 11. The samples after aging are characterized by higher tensile strength with a decrease in their elasticity, which is visible in the results of Young’s modulus and tensile elongation. This is due to the stiffening of the system through the hydrolysis of some residual Si-OEt and some Si-OCH2CH2OC(O)O~ groups and the formation of a densely packed network of Si-O-Si bonds. It is possible that in the case of SPUR-2, the process caused partial or major destruction of the oligomeric structure of the silanizing agent, so the final structure of SPUR-1 and SPUR-2 after water treatment might be similar.

Additionally, thermal properties were determined for both SPUR-cured polymers. The DSC analysis was performed to confirm the amorphous structure and determine their glass transition temperature (T_g_, Figure S3). It can be seen that the T_g_ for the SPUR-1 (T2 silanizing agent) sample is −54.52 °C, and for the SPUR-2 (C9 silanizing agent) sample, it is −56.21 °C. The SPUR-2 sample has both a lower Young’s modulus (higher elongation) and a slightly lower T_g_, which confirms that it is characterized by greater flexibility. The thermal stability of SPUR-1 and SPUR-2 was determined using TGA analysis (Figure S4). No significant differences between the samples were revealed. The products are characterized by thermal stability up to 170 °C. Based on the presented results of the mechanical and thermal analyses, one can conclude that the obtained materials can be successfully applied as flexible sealants with a wide working window from −40 to about 150 °C. 

## 3. Materials and Methods

### 3.1. Materials

APTES (99%, Sigma-Aldrich, St. Louis, MO, USA), EC (99%, anhydrous, Sigma-Aldrich), 1,8-diazabicyclo[5.4.0]undec-7-ene (DBU, 98%, Sigma-Aldrich), dibutyltin dilaurate (Sn(IV), 95%, Sigma-Aldrich), tin(II) 2-ethylhexanoate (Sn(II), ≥92,5%, Sigma-Aldrich), 1,4-Diazabicyclo[2.2.2]octane (DABCO, ≥98%, Sigma-Aldrich), zirconium acetylacetonate (Zr(II), 98%, Sigma-Aldrich), zinc acetylacetonate hydrate (Zn(II), Sigma-Aldrich), triethylamine (TEA, analytical purity, POCh, Gliwice, Poland, triphenylphosphine (Ph_3_P, ≥99%, Sigma-Aldrich), titanium(IV) butoxide (Ti(IV), 98%, Sigma-Aldrich), oleic acid (puriss, Sigma-Aldrich), N,N′-Dicyclohexylcarbodiimide (99%, Sigma-Aldrich), tolylene-2,4-diisocyanate (TDI, technical grade, 80%, Sigma-Aldrich) were used as supplied.

Polyoxypropylene glycol PETOL 56-2 (PETOL, Mn = 2000 g/mol, Oltchim, Ramnicu Valcea, Romania) and diisononyl phthalate (DINP, ester content ≥ 99% (mixture of C9 isomers), technical grade, Sigma-Aldrich) were dried by heating at 90 °C under reduced pressure for 16 h. Toluene (HPLC grade, Sigma-Aldrich), as an internal standard, was dried under reflux with metallic sodium, distilled on molecular sieves 4 Å and stored under nitrogen. Dichloromethane (DCM, HPLC grade, POCh), as a solvent for use without purification, was used as an eluent for GC, GPC and MALDI-ToF. CDCl_3_ (99.8%, Deutero, Kastellaun, Germany), as a solvent ^1^H and ^29^Si NMR analysis, was dried under reflux with CaH_2_, distilled on molecular sieves 4 Å and stored under nitrogen. All substances purchased from Sigma-Aldrich were delivered by Sigma-Aldrich, Poznan, Poland.

### 3.2. Synthesis Methods

#### 3.2.1. Synthesis of Hyperbranched Polymers Terminated with a Hydroxyl Group

In a Schlenk flask equipped with a stirring bar, 1.47 g (22.3 mmol) of EC, 4.94 g (22.3 mmol) of APTES, 0.1 mL of dry Toluene as an internal standard, and optionally, 0.5 wt% of an appropriate catalyst with respect to the sum of EC and APTES, e.g., 35.1 mg (0.134 mmol) of Ph_3_P, were placed under nitrogen to avoid moisture. The mixture was placed in an oil bath at the set temperature, and the reaction was monitored using GC analysis. Additionally, one of the products obtained at the presence of Ti based catalyst was subjected to vacuum distillation in order to remove low-molecular-weight ethanol by-product from the system and to obtain a (hyper)branched condensation polymer. The products of the reactions were determined by ^1^H and ^29^Si NMR, MALDI-ToF, FTIR, and GPC.

#### 3.2.2. Esterification of Oleic Acid with APTES-OH Blocking of Monomer Stability in MALDI-ToF Analysis

In a Schlenk flask equipped with a stirring bar, 1.68 g (5.93 mmol) of oleic acid, 1.22 g (4.0 mmol) of reaction product T2 (consisting mainly of APTES-OH monomer) and 1.22 g of dicyclohexyl carboimide (5,93 mmol) were placed under nitrogen to avoid moisture. The reaction was carried out for 24h at room temperature. The products of the reactions were determined by MALDI-ToF.

#### 3.2.3. Synthesis of Silane-Modified Polyurethane (SPUR)

In a three-necked round-bottom flask equipped with mechanical stirring, a condenser and a gas bladder, 4.2 mL (29 mmol) of TDI, 9.0 mL (21 mmol) of DINP and 30 g (14.5 mmol) of PETOL were placed under a nitrogen atmosphere (the catalyst Sn(IV) was used in the amount of 0.1 wt% with respect to the weight of all substrates without plasticizer). The flask was located in an oil bath at 50 °C for 2 h. The course of the reaction was monitored by FTIR, observing the complete disappearance of the bands originating from the hydroxyl group. An amount of 9 g (29 mmol) triethoxysilane terminated with a hydroxyl group prepared at room temperature and without using a catalyst was added to the reaction flask. The course of the reaction was monitored by FTIR (analysis of samples collected), observing the complete disappearance of the bands originating from the isocyanate group. A reaction was also performed using a hyperbranched condensation polymer as the silanizing agent. After the reactions were complete, the crosslinking catalyst (Sn(IV)) was added to the reactions flask in an amount of 1 wt% with respect to the weight of all substrates without plasticizer. The next step was to pour the mixture onto a Teflon fabric in order to obtain a joint based on SPUR. The products of the reactions were determined by FTIR, DSC and TGA. Additionally, the mechanical properties of the obtained polymer films were determined.

### 3.3. Measurements

^1^H NMR and ^29^Si NMR analysis was performed on Varian NMR System 500 MHz spectrometer (Palo Alto, CA, USA) using dry CDCl_3_ as a solvent. GC analysis was carried out using the Agilent 7820A GC System equipped with an HP-5 column, used to study the conversion of EC. The molar mass and molar mass distribution were determined by GPC on a Viscotek system comprising GPCmax and TDA 305 (triple detection array (TDA): RI, IV, LS) equipped with DVB Jordi gel column(s) (linear, mixed bed) in DCM as an eluent at 30 °C at a flow rate of 1.0 mL/min. MALDI-ToF mass spectrometry was performed on Bruker Daltonics UltrafleXtreme™ (Billerica, MA, USA). Trans-2-[3-(4-tert-butylphenyl)-2-methyl-2-pro-penylidene] malononitrile (DCTB) was used as the MALDI matrix. FTIR spectra were recorded on a Thermo Scientific Nicolet iS5 spectrometer. Tensile strength tests were carried out using Instron 5566 testing machine equipped with a 100 N measuring head and pneumatic grips. The measurement was performed at room temperature with a running rate of 200 mm/min. Tensile strength test species in the shape of “dog bone”. A total of 10 samples before aging and 3 samples after aging were tested. Data were processed with BlueHill2 software. DSC measurements were taken with a TA Instruments Q2000 calorimeter. Polymer samples (in the amount of about 10 mg) were subjected to time-tested temperature profiles: heating I from 25 °C to 200 °C at a rate of 10 °C/min, cooling from 200 °C to −80 °C at a rate of 20 °C/min and heating II from −80 °C to 200 °C at a rate of 20 °C/min. TGA measurements were recorded with a TA Instrument SDTG600. Polymer samples in the amount of approx. 10 mg were heated in the temperature range from 10 °C to 1000 °C, with a heating rate of 10 °C/min. The process was carried out in two variants: with air and in an inert gas atmosphere (Ar).

## 4. Conclusions

In this work, the reaction of the APTES primary amino group with a five-membered EC was used to obtain hydroxyl-terminated alkoxysilane monomer and oligomers. The resulting APTES-OH product, containing in its structure a primary hydroxyl group and ethoxysilane groups, can undergo a self-polycondensation process to form hyperbranched structures under favorable conditions. The influence of temperature and catalysts on the degree of polymerization of hydroxy-terminated alkoxysilane oligomers was investigated. It was shown that the optimal conditions for the synthesis of APTES-OH monomer with high selectivity are the reaction at a temperature of 20–25 °C without the addition of a catalyst. On the other hand, in order to obtain the APTES-OH oligomers with the greatest efficiency, the reaction should be carried out at an elevated temperature (50–90 °C) with the use of a catalyst, most favorably based on Ti(IV). The intensification of oligomers synthesis is also favored by supplying reduced pressure in order to remove the condensation by-product. The MALDI-ToF and GPC analyses confirmed the attainment of oligomers with the number of repeat units up to 20–25. Additionally, the ^29^SiNMR analysis showed the formation of a branched structure. Urethane prepolymers were successfully modified chemically by reaction with APTES-OH and its oligomers. The mechanical and thermal properties of the obtained materials were examined, and their aging tests in water were also carried out. The tensile strength value reached slightly more than 500 kPa in the case of both samples studied, whereas the elongation at break was almost 30% higher for the SPUR-2 sample (C9 oligomeric silanizing agent) when compared to SPUR-1 (T2 monomeric silanizing agent). This gives the conclusion that higher oligomerization and branching degree of the presented silanizing agent led to improvement in flexibility and extensibility of SPUR at no significant change in tensile strength. The samples after aging are characterized by higher tensile strength with a decrease in their elasticity. This is due to the stiffening of the system through reactions of all Si-OEt groups in the structures, and thus, the formation of a densely packed network of Si-O-Si bonds. The obtained materials are amorphous and have a similar value of the glass transition temperature (around −54 °C) and are characterized by thermal stability up to 170 °C. The tested types of SPUR can be successfully used as an adhesive or elastic sealant with a wide working window from −40 to about 150 °C.

## Figures and Tables

**Table 1:** Conditions and catalysts used in the reactions of EC with APTES.

No.	Temperature [°C]	Catalyst	Time [h]
T1	20	-	24
T2	25	-	23
T3	30	-	23
T4	50	-	22
T5	70	-	25
T6	90	-	23
T7	110	-	25
C1	30	DBU	4
C2	30	Ph_3_P	3
C3	50	DBU	3
C4	50	Ph_3_P	3
C5	50	Sn(II)	3
C6	50	Sn(IV)	3.5
C7	50	DABCO	3
C8	50	Ti(IV)	3
C9 ^a^	50	Ti(IV)	3 + 3.5 ^b^
C10	50	Zn(II)	4
C11	50	Zr(IV)	4.5
C12	50	TEA	4.5

EC: APTES molar ratios were 1:1. The weight fraction of the catalyst in the reaction was 0.5%. The reactions were carried out until the conversion of EC reached 95–99%. The reaction progress was followed by means of GC. ^a^ Product was subjected to vacuum distillation in order to remove ethanol from the system. ^b^ Reaction was carried out for 6.5 h, including 3 h at atmospheric pressure and 3.5 h under diminished pressure (vacuum distillation).

## Data Availability

The data presented in this study are available on request from the corresponding author.

## Supplementary Materials

The following supporting information can be downloaded at: https://www.mdpi.com/article/10.3390/molecules27092790/s1, Figure S1: Structures of the obtained populations in the MALDI-ToF analysis and the tested structure possibilities for an undefined population; Figure S2: FTIR spectrum of the SPUR-2 reaction; Figure S3: DSC curves of the second heating cycle for products SPUR1 and SPUR2; Figure S4: TGA analysis for products SPUR1 and SPUR2; Table S1: Molar masses of repeating units and end groups of identified and proposed structures; Table S2: Tensile strength results of the SPUR products.

## Sample Availability

Samples of all compounds are available from the authors, after re-synthesis.
