# Supplementary material for: Synthesis of (Hyper)Branched Monohydroxyl Alkoxysilane Oligomers toward Silanized Urethane Prepolymers
Source: Molecules. 2022 Apr 27;27(9):2790. doi: 10.3390/molecules27092790 (PMC9105832; doi:10.3390/molecules27092790)
Supplement: Supplementary file 1 [file molecules-27-02790-s001.zip › molecules-1670118-supplementary.pdf]

Supporting Information

Synthesis of (hyper)branched monohydroxyl alkoxysilane oligomers toward silanized urethane prepolymers

Sebastian Kowalczyk [1], Maciej Dębowski [1], Anna Iuliano [1], Sebastian Brzeski [1], Andrzej Plichta [1]

[1] Chair of Chemistry and Technology of Polymers, Faculty of Chemistry, Warsaw University of Technology, Noakowskiego 3, 00-664 Warsaw, Poland

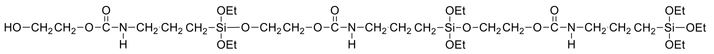

S1.1

Main product of condensation

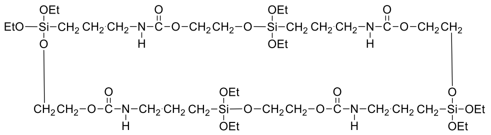

S1.2

Product of intramolecular condensation

S1.3

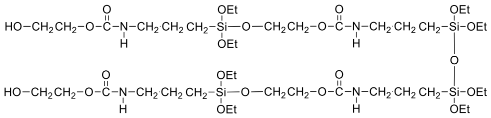

S1.4

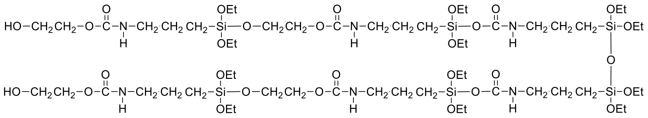

S1.5

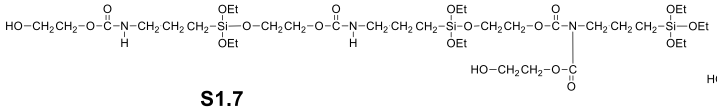

S1.6

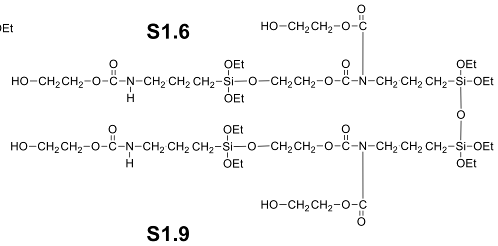

S1.7

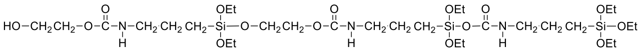

S1.8

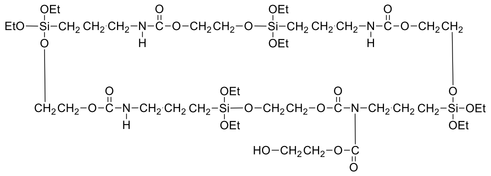

S1.9

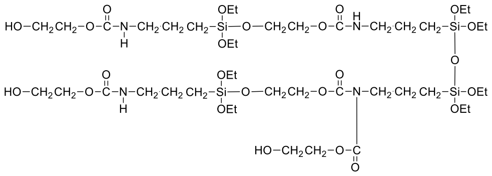

The proven possibilities of the structure

Figure S1. Structures of the obtained populations in the MALDI-ToF analysis and the tested structure possibilities for an undefined population.

Table S1. Molar masses of repeating units and end groups of identified and proposed structures.

Identified structures		
Name	Molar mass of repeating unit [g/mol]	Molar mass of end group [g/mol]
**S1.1**	263.1	46.51
**S1.2**	263.1	0.37
**Proposed structures**		
**S1.3**	263.1	18.09
**S1.4**	263.1	193.17
**S1.5**	263.1	134.11
**S1.6**	263.1	194.12
**S1.7**	263.1	2.07
**S1.8**	263.1	88.09
**S1.9**	263.1	106.10

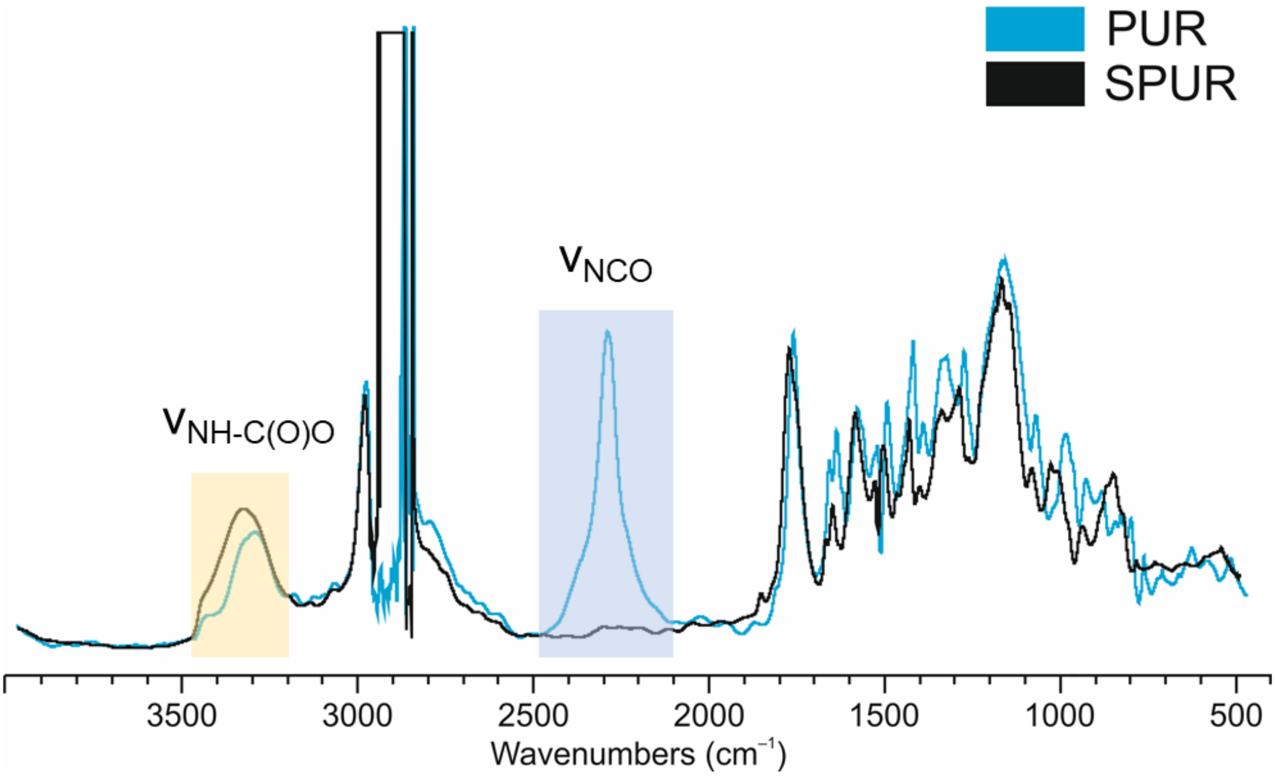

Figure S2. FTIR spectrum of the SPUR-2 reaction.

Table S2. Tensile strength results of the SPUR products.

No.	Silanizing agent	Young's modulus [MPa]		Tensile Strength [MPa]		Elongation at Break (%)	
		x̅	s.d.	x̅	s.d.	x̅	s.d.
SPUR-1	T2	0.408	0.009	0.527	0.036	178.3	16.3
SPUR-1*		1.103	0.053	0.882	0.045	97.2	3.4
SPUR-2	C9	0.315	0.007	0.563	0.024	229.7	11.8
SPUR-2*		0.850	0,083	0.718	0.153	98.7	14.4

* the samples aged for 24 days in water

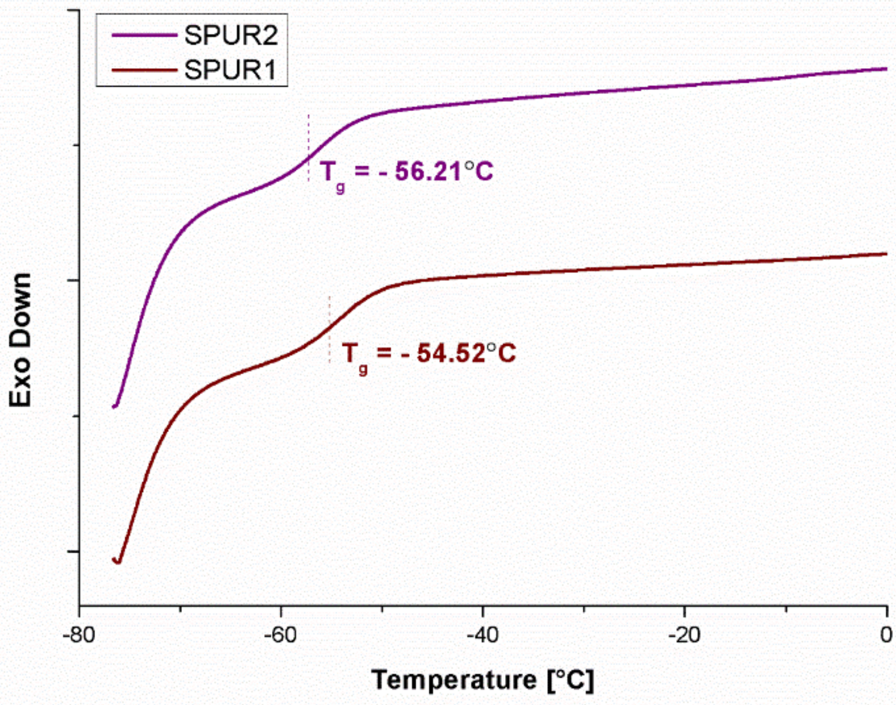

Figure S3. DSC curves of second heating cycle for products SPUR1 and SPUR2.

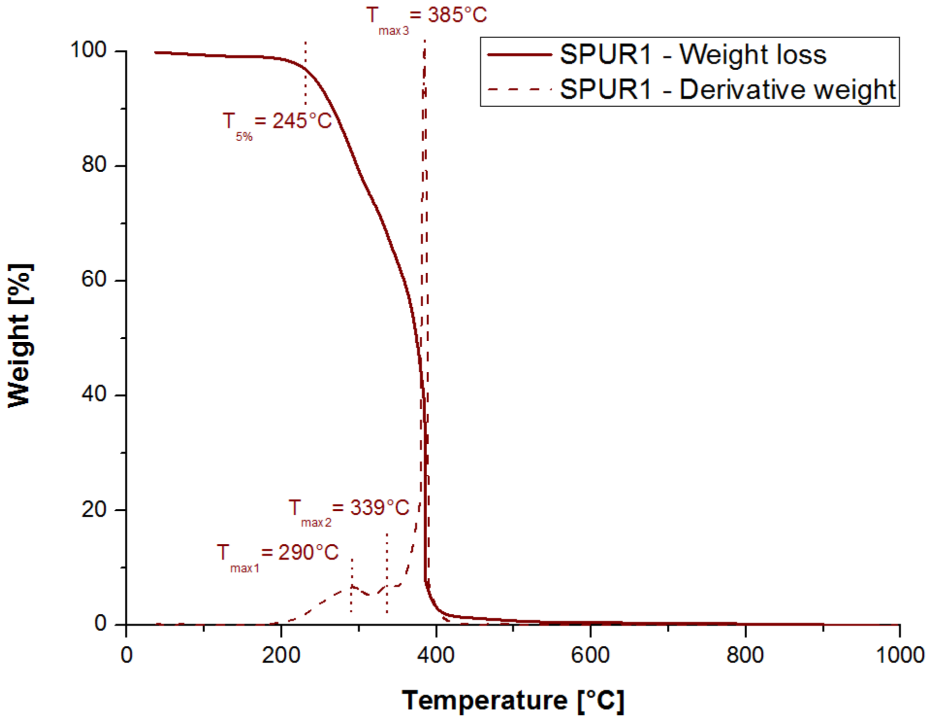

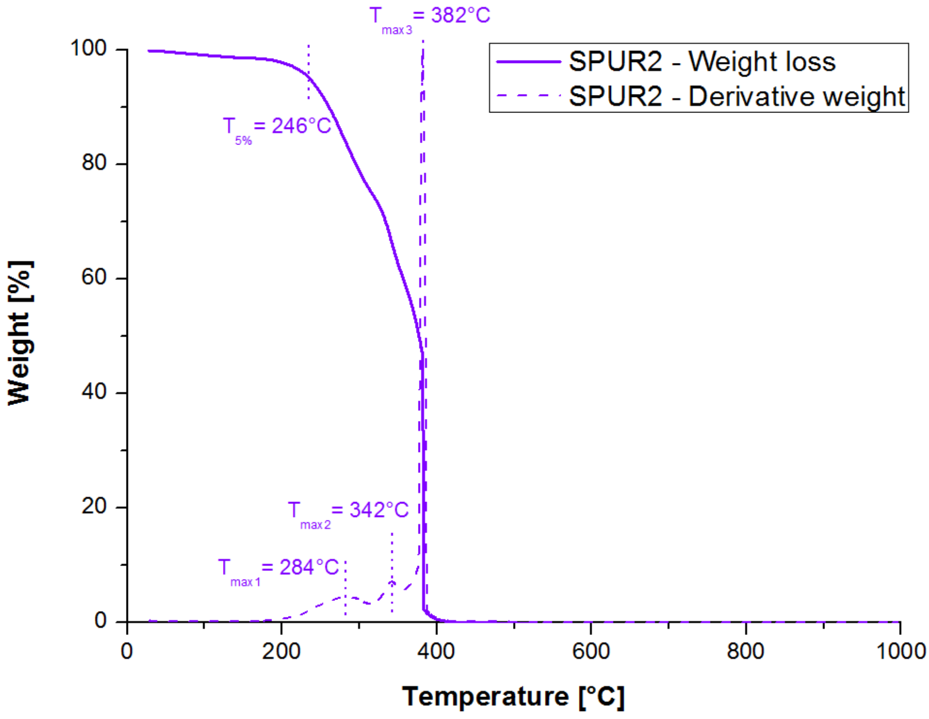

Figure S4. TGA analysis for products SPUR1 and SPUR2.
